# An Omics Approach to Diagnosing or Investigating Fungal Keratitis

**DOI:** 10.3390/ijms20153631

**Published:** 2019-07-25

**Authors:** Ming-Tse Kuo, Jiunn-Liang Chen, Shiuh-Liang Hsu, Alexander Chen, Huey-Ling You

**Affiliations:** 1 Department of Ophthalmology, Kaohsiung Chang Gung Memorial Hospital and Chang Gung University College of Medicine, Kaohsiung 83301, Taiwan; 2 Department of Ophthalmology, Kaohsiung Veterans General Hospital, Kaohsiung 81362, Taiwan; 3 Department of Ophthalmology, Chung-Ho Memorial Hospital, Kaohsiung Medical University, Kaohsiung 80756, Taiwan; 4 Department of Laboratory Medicine, Kaohsiung Chang Gung Memorial Hospital and Chang Gung University College of Medicine, Kaohsiung 83301, Taiwan

**Keywords:** fungal keratitis, molecular diagnosis, genomics, metagenomics, proteomics

## Abstract

Fungal keratitis (FK) is one of the most severe corneal infectious diseases. FK often leads to poor visual prognosis and thus requires accurate diagnosis. Conventional approaches, including clinical diagnoses, smears, and cultures, often fail to provide reliable diagnostic value. Omics approaches, such as those using genomic, metagenomic, and tear proteomic data sources, provide promising features for improving the diagnosis and monitoring the progression of FK. Genomic approaches are based mainly on detecting amplicons of ribosomal RNA genes, and internal transcribed spacers are gradually gaining popularity in clinical practices. A metagenomic approach based on 16S rRNA genes may help monitor the dynamic change of conjunctival microbiota associated with an FK event, whereas that based on shot-gun and 18S rRNA target enrichment sequencing could have the potential to diagnose FK using clinical samples. A tear proteomic approach may provide comprehensive information about ocular surface defense and injury during FK. Representative up- and down-regulated proteins during FK could also be used as biomarkers to determine the clinical course and develop a treatment strategy in different stages of FK. Consequently, a personalized tear proteomic approach will soon play a key role in FK management.

## 1. Introduction

Fungal keratitis (FK) is one of the most devastating microbial keratitis with the worst visual prognosis, potentially leading to blindness [1,2]. Over 40% of microbial keratitis cases are caused by fungal infection in several tropical and subtropical countries [3,4,5,6]. The clinical characteristics of FK are mild pain, the insidious growth of fungal pathogens in the deep cornea, and difficulty in differentiating it from other kinds of microbial keratitis early on. Therefore, the early and accurate diagnosis of FK by means of clinical features is sometimes impossible due to patients’ delayed visits or the great similarities of FK with other types of microbial keratitis in early stages of the disease. In addition, conventional laboratory approaches using smears (direct microscopy) and microbial cultures fail to serve as reliable diagnostic tools in many cases. Direct microscopy is limited by examiners’ experience, while the culture approach is time-consuming and incapable of isolating fastidious fungal pathogens. As a result, several severe complications, such as hypopyon, glaucoma, iris atrophy, cataract, corneal melting, corneal perforation, and endophthalmitis [6,7], may occur in patients with FK.

In addition to the problems of current clinical and laboratory diagnosis, the medical treatment of FK is also full of challenges. Approximately one third of FK patients were refractory to antifungal agents and ultimately required therapeutic keratoplasty [8,9]. Currently available antifungal agents have limitations not only in the drug-dependent capacities of corneal penetration, but also in species-dependent fungicidal capacity. Tracing the predictors of medical treatment failure, including old age, trauma, large lesions, deep infiltrates, positive fungal culture results, day 6 repeated fungal culture positive results, *Aspergillus* species isolation, and higher minimum inhibitory concentrations (MICs) to natamycin [10,11,12], identification of the fungal pathogen plays a critical role in adopting surgical management in time. 

In order to rescue the vision of those patients with a delayed visit, minimize the risk of medical treatment failure, and quickly determine the need for surgical management, precise diagnosis is the decisive factor. Omics technology advancements, such as genomics, proteomics, and metabolomics, have been exploring the biochemical changes in ocular surface samples of FK over the past two decades. Through the collective characterization and quantification of pools of biochemical molecules, omics technologies provide several alternative methods for diagnosing and monitoring FK. Among these technologies, genomics approaches have been widely proven to be good alternatives of conventional smear and culture methods, while proteomic and metagenomic FK also show promising results in some studies.

The characteristics of ocular surface samples are critical considerations in diagnosing FK because the number of ocular surface samples is low, and collected samples usually carry many fewer pathogens than non-ocular samples. Therefore, we first reviewed the characteristics of FK, the clinical diagnosis of FK, the conventional and non-omics laboratory diagnosis techniques of FK, followed by useful ocular samples for an omics approach, and finally focused on an omics approach in the aid of current clinical practice and investigation of FK.

## 2. Literature Search

**Figure 1:**
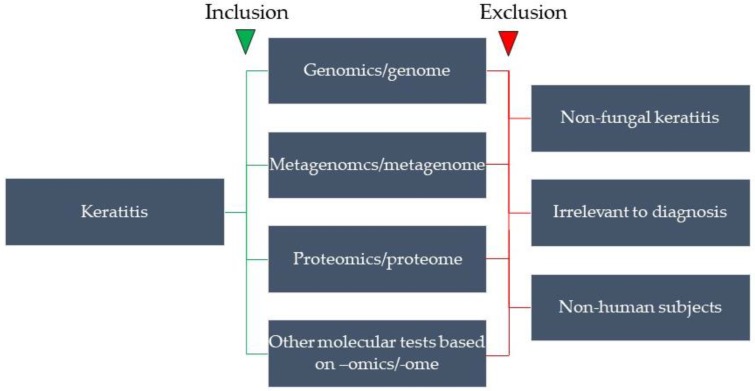
The inclusion and exclusion criteria of the PubMed search in this review.

We searched the PubMed database for articles published in English on the omics approach to FK. The search strategy included the following keywords, “fungal keratitis”, “mycotic keratitis”, “keratomycosis”, “diagnosis”, “genome”, “genomics”, “polymerase chain reaction”, “oligonucleotide hybridization”, “next-generation sequencing”, “metagenome”, “metagenomics”, “transcriptome”, “transcriptomics”, “metatranscriptome”, “metatranscriptomics”, “mass spectrum”, “mass spectrometry”, “proteome”, “proteomics”, “metaproteome”, “metaproteomics”, “lipidome”, “lipidomics”, “glycome”, “glycomics”. The anterior 3 keywords were variously combined with the following keywords for a specific search. The articles were selected based on the following inclusion and exclusion criteria (Figure 1).

## 3. Pathogens and Risk Factors of FK

Common fungal pathogens are frequently morphologically classified into filamentous and yeast-like fungi [13], with filamentous fungi being more common than yeast-like fungi worldwide. The fungal pathogen of FK is generally opportunistic corneal infection predisposed to corneal surface trauma [9]. According to the result of the Asia Cornea Society Infectious Keratitis Study [14], FK was the second common microbial keratitis with slightly fewer cases than bacterial keratitis (BK) (FK: BK = 33%: 38%). However, among the 2831 microorganisms isolated from patients with suspected microbial keratitis, the top 3 pathogens were *Fusarium* spp. (18%), *Pseudomonas* spp. (10%), and *Aspergillus* spp. (8%). In addition to *Fusarium* and *Aspergillus*, *Curvularia* spp., *Alternaria* spp., and *Candida* spp. were also commonly reported pathogens of FK [15,16,17,18].

Climate also plays a major role in pathogen determination. The proportions of common pathogens of FK in temperate regions are different from those in tropical and subtropical regions. For example, in the temperate climate of a Danish population, 52% FK patients were infected with *Candida*, 20% with *Fusarium*, 16% with *Aspergillus,* and 12% with mixed filamentous fungi [19].

The major risk factors of FK include a tropical climate, the rainy season, trauma, being an agriculture worker, and a rural area [3], whereas the other risk factors are associated with urbanization, including contact lens wear, ocular surface disease, and immunocompromised status, such as diabetic mellitus and corticosteroid exposure [16,19,20]. Although tropical climate is a major risk of FK, increased incidences have been reported in temperate climate regions in recent years [3,21].

## 4. Clinical Diagnosis of FK

Corneal ulceration features of FK identified by slit lamp biomicroscope include feathery/serrated/irregular margin, raised slough, dry texture, satellite lesions, and colorization. Among these features, the presentation of a feathery margin, raised slough, and colorization were significantly and independently associated with FK. However, the feathery margin, agreed by most ophthalmologists, is the hallmark that distinguishes FK from BK [22,23]. In addition to corneal ulceration, corneal endothelial plaque and hypopyon were sometimes present in FK patients with deep corneal invasion. Even with the above clinical morphologies, the overall diagnostic accuracy is less than 70%, even when impressed by a corneal specialist [23].

In vivo confocal microscopy is a novel tool to non-invasively diagnose FK in a clinical office [24]. Under in vivo confocal microscopy, the presentation of highly reflective lines with numerous interlocking branches could be identified as filamentary FK, whereas the exhibition of hyper-reflective deposits or pseudofilaments could be recognized as yeast-form FK [25]. However, diagnostic accuracy is highly dependent on observers’ experiences, with only moderate sensitivity (71.4%), even in experienced observers [25].

## 5. Laboratory Diagnosis of FK

Microbial culture via corneal scraping samples is still the current gold standard for diagnosing FK, while a smear by a KOH wet mount or a gram stain was recommended as an additional clinical routine for rapidly confirming FK [26,27,28,29]. Microbial culture can identify fungal species and facilitate an antifungal susceptibility test, but it is time-consuming and fails to detect fastidious fungal pathogens [27,30,31]. On the other hand, direct microscopic examination is currently the fastest diagnostic method, given that light microscopy and associated reagents are equipped in the clinical office. However, the results of direct microscopy for FK have highly variable sensitivity, possibly due to variation in examiners’ experiences [26,27,29,31]. In a four case preliminary study [32], measurement of the tear β-D-glucan level was reported to have a high sensitivity for diagnosing FK, but there is no further study to validate such a finding. Fortunately, molecular tests based on different omics approaches for diagnosing FK are gaining popularity in recent years and show great promise in routine clinical practices. Therefore, we will summarize and discuss the progresses of omics approaches for diagnosing and investigating FK in the following section.

## 6. Samples for Omics Approaches

### 6.1. Clinical Isolates

Pathogens of FK may be isolated from microbial cultures of ocular surface samples, which are obtained via direct scraping of the corneal ulcer, swabbing of the corneal ulcer site, and collecting of the seeding microbes from the tears. The culture media for obtaining fungal isolates include solid and liquid media. Blood agar and chocolate agar were the standard solid media for BK and were able to recover about 56%–79% and 44%–53% of fungal pathogens, respectively [27,30]. Sabouraud dextrose agar was thought to be the standard culture medium for FK, but this remains controversial [27,30]. Researchers have suggested omitting this agar due to its time-consuming isolation of fungal pathogens and an even lower recovery rate than blood agar [30]. On the contrary, supporters recommended using this agar for FK due to its ability to identify pathogens, its *Histoplasma* recovery, and its acceptable recovery rate [27]. Although liquid culture media can increase the chance of isolation of mixed BK and FK, their role in isolating pure FK is limited [33]. For maximal recovery of clinical isolates, inclusion of both solid and liquid culture media is recommended.

### 6.2. Corneal Scrapes

Corneal scraping should be performed by either a sterile Kimura spatula or a 15# surgical blade under a biomicroscope with a sterile procedure to prevent flora contamination from either the conjunctiva or eyelid [34]. The sampling area should include not only the margin of the ulcer, but also the stroma of the ulcer center. Some fungal pathogens may invade deeper stroma, and a false negative result of the laboratory tests may occur if only superficial samples from the margin of the ulcer are obtained. For deeply invaded FK, a corneal biopsy or keratoplasty must be considered to obtain the infectious corneal tissue, especially for those with a medically refractory course. For FK breaking through the cornea into the aqueous chamber, aqueous tapping may be considered to obtain aqueous samples before anterior chamber irrigation with an anti-fungal agent.

### 6.3. Corneal Swabs

Corneal swabbing with a transportation medium may be used initially to minimize the destruction of corneal stromal tissue. Culture results using the single-swabbing approach were more accessible and less cumbersome than corneal scraping and are comparable to the multi-sample method [35]. Subsequent inoculation to the indicated culture media can be assisted by technicians in a clinical microbial laboratory. Therefore, the swabbing approach is suitable for patients with small and superficial ulcers, as well as for ophthalmologists in the community setting who do not have access to the full set of culture media.

### 6.4. Tears

Tears may serve as a reservoir for fungal pathogens and may reflect the ocular surface environment altered by FK. Tear sampling consists of mainly the following three types: basal tears, reflex tears, and flush tears [36]. Basal tears are gently obtained by microcapillary tubes to prevent any stimulus to the ocular surface from the tear reflex. Thus, the sample amount is the most scarce among the other tear samples. On the contrary, reflex tears are collected under various stimuli such as light, odor, emotion, and nasal irritation. Reflex tears are mainly from tears secreted by lacrimal glands, so tear components released from the ocular surface may be diluted. The third kind of tear sample is flush tears [37]. This tear sample is obtained by first instilling a certain amount of normal saline or balance salt solution to wash the ocular surface and then collecting the fluid composed of tears and post-instillation solutions. By comparing the above tear samples, basal tears are best suited for investigating normal subjects with enough basal tears and for analyzing tear lipids, as the reflex and flush tears contain very low levels of most lipid components [36]. For FK, reflex tears may be naturally obtained from the ocular surface in patients with severe ulceration [38,39]. Flush tears are more suitable than basal tears for analyzing tear proteins [40] and are the best choice for collecting tears in patients with severe tear deficiency or poor reflex tears, such as Sjögren syndrome patients [41]. Moreover, flush tears sampling could be used for a standardized approach because flush tears can be obtained from different severities of FK.

## 7. Diagnosing or Investigating FK by an Omics Approach

We summarized the representative studies about omics approaches associated with the diagnosis and investigation of FK in the past two decades in Table 1.

### 7.1. Genomics

Genome-based tests for diagnosing FK (Table 1) are highly sensitive, less time-consuming than cultures, and are ideal for ocular surface samples with a low number of samples. The target DNA of FK detection include highly conserved ribosomal RNA (rRNA) genes (18S, 5.8S, and 28S rRNA genes), internal transcribed spacers (ITSs 1 and 2), elongation factor 1—alpha gene [47], and the mitochondrial cytochrome b gene [52]. Among the target DNA, the amplicons of the polymerase chain reaction (PCR) amplified from the 18S rRNA gene were most commonly used for detecting fungal infection. ITSs 1 and 2 have a high copy number the same as rRNA genes but have a high degree of variation among species. Therefore, amplicons with ITS regions can be used to identify fungal species by DNA sequencing [58], multiplex PCR [53], or hybridization [59]. PCR amplification for gel electrophoresis is now a common molecular technique and can be performed by laboratory technicians. However, the detection limit of PCR is poorer than that of other molecular tests, and nested or semi-nested PCRs may be needed to increase the sensitivity [42,43,46,60]. Sanger technology is limited by the short-read sequencing. With the great development of high-throughput next-generation sequencing platforms and technologies, DNA sequencing for ITS barcoding is no longer done in specialized sequencing centers and reference research laboratories only [61].

Real-time PCR is a highly efficient molecular technique in diagnosing FK, with a turnaround time of about 2.5 h [47], but this technique needs a sophisticated instrument. Real-time PCR with a high resolution melting analysis (PCR-HRM) can detect fungi, differentiate yeasts from filamentous fungi, and discriminate among relevant species of yeasts simultaneously [49]. Dot hybridization assay (DHA) is a highly sensitive technique [50] with the capacity to develop an oligonucleotide array for identifying fungal pathogen to species level. Nonetheless, FK has a wide spectrum of fungal pathogens, which are mainly composed of opportunistic infections by means of ocular trauma. The reasonable fungal species targets for designing oligonucleotide probes of DHA should be common fungal species, especially those frequently refractory to anti-fungal agents. Direct PCR adopted a special buffer and DNA polymerase without the step of DNA extraction for directly amplifying the fungal DNA template from corneal scraping samples [51]. Therefore, the turnaround time of complete procedures was decreased to 3 h. In summary, the sensitivities of most genome-based tests for diagnosing FK were reported to be near 90% or higher, but the specificities of these techniques were highly variable, ranging from 17% to 97%. The low specificities of these studies may be due to the assumption that PCR-positive but culture-negative samples were false positives or that the sample was contaminated by the environment during either sampling or laboratory processing.

### 7.2. Metagenomics

Shigeyasu et al. reported a difficult-to-identify and medically refractory case of FK, confirmed by metagenomic analysis with corneal scraping and aqueous fluid samples [54]. The authors adopted metagenomic shotgun next-generation sequencing by a MiSeq platform (Illumina, Inc., CA, USA) and a cloud-computing pipeline MePIC for pathogen identification. With this technique, DNA is randomly broken up into small segments, which are sequenced using the chain termination method to obtain reads. After genomic subtraction from the original 993,734 reads of the corneal scraping sample, 7977 reads remained after removing ambiguous and host-derived short reads; 52.4% (4177/7977) reads were derived from eukaryota, and 22 reads had a high similarity with *Nectria haematococca*, which belongs to the *F. solani* species complex. From the sample collected from anterior chamber fluids, the original 1,115,468 reads were obtained, and 12,232 reads remained for a further sequence alignment with the database. Accordingly, 38.3% (4684/12,232) of reads were derived from eukaryota, and four reads had a high similarity with *N. haematococca*, which was consistent with the result of the corneal scraping sample. The analysis of metagenomics was compatible with the result of genomic DNA sequencing (ITS1, 5.8S rRNA gene and ITS2), of which *F. solani* was identified by the BLAST database from the isolate recovered by potato dextrose agar. The advantage of metagenomic analysis is to identify the unbiased sequencing of host and pathogens within clinical samples [62]. Compared with genomics-based tests, the metagenomic analysis is also suitable for identifying unculturable, slow-growing, and fastidious pathogens. Although the cost of next-generation sequencing was gradually reduced, the data preprocessing for metagenomic analysis is too heavy to routinely perform this examination. A standardized metagenomic analysis pipeline approach for removing ambiguous and host-derived short reads and identifying disease-causing pathogens rapidly in FK patients should be considered. In addition, target enrichment next-generation sequencing for fungal ITS region may provide a more reliable result by capturing the target genes and removing non-target genes before sequencing [63].

Ge et al. tried to elucidate the alteration of conjunctival microbiota for patients with FK by using high-throughput 16S rRNA gene sequencing metagenomic analysis to compare conjunctival swab samples from the healthy eyes, fellow eyes, and infected eyes of FK [55]. An operational taxonomic unit (OTU) was used as the analytical unit of microbial diversity for high-throughput rRNA gene sequencing. The author found that infected and fellow eyes had reduced the bacterial diversity of ocular surface microbiota compositions with lower abundance of *Corynebacterium* and *Staphylococcus* and higher abundances of *Pseudomonas*, *Achromobacter*, *Caulobacte*r, and *Psychrobacter* according to the analyses of alpha diversity, beta diversity, and dominant genus analysis. The authors concluded that these changes may contribute to the pathogenesis of FK or an increased risk for FK. *Pseudomonas* was also mentioned to have an anti-fungal activity through its secondary metabolites, but the cause of its overgrowth in FK patients is still unclear. Kalyana Chakravarth et al. analyzed fungal and bacterial microbiota in fecal specimens through the high-throughput sequencing of the ITS 2 region for fungi and the V3–V4 region of the 16S rRNA gene for bacteria in healthy controls and patients with FK [56]. The alpha diversity of the gut bacterial microbiota was measured in terms of the number of observed OTUs per specimen (richness), the Simpson index (evenness), and Shannon diversity. The author found *Candida albicans* (2 OTUs), *Aspergillus* (1 OTU), and three other denovo-OTUs were enriched in FK samples and an unclassified denovo-OTU was enriched in the control samples. However, the overall abundances of these identifiable OTUs were very low. This result was not indicative of significant dysbiosis in the fungal microbiota inhabiting the bowels of FK patients. Similar to conjunctival microbiota alteration in FK [55], the gut bacterial diversity in FK patients was significantly decreased when compared to normal controls. Fifty-two OTUs were significantly increased in normal subjects, but only 5 OTUs were increased in FK patients. The enriched OTUs in normal subjects were identified as *Faecalibacterium prausnitzii*, *Bifidobacterium adolescentis*, *Lachnospira*, *Mitsuokella multacida*, *Bacteroides plebeius*, *Megasphaera*, and *Lachnospiraceae*. In FK subjects, 5 OTUs linked to *Bacteroides fragilis*, *Dorea*, *Treponema*, *Fusobacteriaceae*, and *Acidimicrobiales* were significantly higher in abundance. The author concluded that the decreased abundance of beneficial bacteria and increased abundance of pro-inflammatory and pathogenic bacteria in FK subjects may contribute to the clinical presentation in FK. In addition, functional studies with specific bacteria altered in the gut microbiome of FK patients could identify the role of these bacteria or their products in the pathogenesis of FK. However, in our clinical practice, a combination of antibiotics and antifungal agents is commonly implemented to prevent secondary bacterial infection. Therefore, instilling topical empirical or prophylactic antibiotics in FK patients may be another cause of alterations in the bacterial diversity of the ocular surface and gut microbiota, which was not mentioned in the above studies.

### 7.3. Proteomics

Tear proteins are produced from the main and accessory lacrimal glands, as well as ocular surface epithelial cells. Ananthi et al. collected reflex tears from culture-proof FK patients and normal subjects for tear proteomic analysis [38]. The author used pooled tears of FK and normal groups for two-dimensional polyacrylamide gel electrophoresis (2D-PAGE) to separate the tear proteins, respectively. The protein dots with different expressions between FK tears and control tears were then identified by matrix-assisted laser desorption/ionization-time of flight mass spectrometry (MALDI-TOF MS). Glutaredoxin-related protein was expressed only in the tears of FK patients, and this protein is known to be produced by *Aspergillus* under oxidative stress conditions. For six abundant tear proteins presented in both groups, prolactin inducible protein and serum albumin precursor were upregulated, whereas cystatin S precursor, cystatin SN precursor, cystatin, and lipocalin were downregulated in the FK group. Therefore, tears may be used as a clinical source to investigate FK, and the tear proteins mentioned above could be used as biomarkers to diagnose or monitor patients with FK.

Ananthi et al. further collected the tear samples of healthy subjects and *Fusarium* keratitis patients in different clinical courses for proteomic analysis [39]. The pooled tear group included normal subjects, early, intermediate, and late *Fusarium* keratitis. To improve protein segmentation and evaluate low-abundant proteins, the author used a two-dimensional difference gel electrophoresis (2D-DIGE) instead of 2D-PAGE and then adopted liquid chromatography-tandem mass spectrometry (LC-MS/MS) for further protein separation and identification. 2D-DIGE circumvents the main drawbacks associated with 2D-PAGE due to low sensitivity, reduced dynamic range, and gel-to-gel variability, enabling more accurate and sensitive protein quantification. Differential regulation of tear proteins at different stages of *Fusarium* keratitis was found. Severe proteins, including α-1-antitrypsin, haptoglobin α2 chain, zinc-α-2-glycoprotein, apolipoprotein, albumin, haptoglobin precursor—β chain, and lactoferrin, were progressively upregulated as the disease progressed towards late stages of *Fusarium* keratitis. In the early stage, the expression of lacritin was reduced to a negligible level compared to the control. The level of cystatin SA III and lipocalin was down-regulated during the late stage of *Fusarium* keratitis. The author used the signals of western blot analysis to validate the up- or down-regulations of three representative proteins (haptoglobin, lacritin, and lipocalin).

Kandhavelu et al. separated tear proteins by glycosylation, 1D-PAGE, and in-gel digestion for LC-MS/MS tear protein identification [57]. The author compared the tear protein profile of pooled tears from early *Aspergillus* keratitis with those from normal subjects. They found that complement system proteins, proteins specific for neutrophil extracellular traps, and proteins involved in wound healing were identified only in the tears of patients. Thus, the early appearance of host defense proteins and a wound healing response indicates that tear proteins could be used as markers for monitoring the progression of *Aspergillus* keratitis. We believe that tear proteomic analysis in FK is a promising approach for investigating the disease’s course. However, a personalized tear proteomic approach [41] will be mandatory for diagnosing FK and monitoring the disease course of FK.

## 8. Other FK-Associated Studies by an Omics Approach

### 8.1. Pathogenic Mechanisms of FK

The pathogenesis of FK, which depends on the fungus–host interaction and the virulence of the invading fungus, is complicated and not well-understood. Although work in this field is relatively insufficient, proteomic approaches may provide valuable information to supplement our current knowledge of FK. Identification of the virulent factors and host responses to the invading fungus are critical to understand the disease’s course and to develop effective treatment strategies. Global genomics, metagenomic, and proteomic approaches are necessary because they are important tools for identifying the pathogenesis of novel targets, which are essential for the intervention and prevention of corneal damage caused by these virulent fungal pathogens.

Calvillo-Medina et al. tried to analyze *F. falciforme’*s ability to form biofilms in vitro and investigate its protein expression, which was isolated from the corneal scrape of an FK patient [64]. They adopted 2D-PAGE separation for MALDI-TOF protein identification and found 19 proteins that were overexpressed in biofilms, compared to planktonic cultures, and six proteins with unique expression in biofilms. Transketolase, enolase, phosphoglycerate kinase, and ATP-citrate synthase were found to be relatively abundant. Some of these proteins in biofilm formation are involved in basal metabolism and have been described as potential virulence factors in fungal infection, whereas the specific roles of these proteins in *F. falciforme* biofilm formation have not yet been determined in this study. Antibiosis interaction of bacterial and fungal pathogens is another issue because mixed infection may occur in a microbial keratitis patients. Therefore, we believe that mixed biofilm formation [65] will be an interesting issue for proteomic analysis in the future.

### 8.2. Assessment of Antifungal Effects on FK

An antifungal susceptibility test increased the odds of successful treatment in FK by a better selection of antifungal agents for these patients, and it is especially important in helping physicians to manage difficult-to-treat FK. By analyzing the prognostic predictors of FK, positive culture result, identification of *Aspergillus* spp., and high MIC to natamycin resulted in a poor visual outcome [11,66]. The gene of antifungal resistance was frequently reported in *Aspergillus* spp. [67,68], which partially explains why *Aspergillus* keratitis was associated with a medical refractory event. In the result of Mycotic Ulcer Treatment Trial I (MUTT I) [11], the authors found that a two-fold increase in MIC was significantly associated with a larger 3 month infiltrate/scar size and increased odds of perforation. For natamycin-treated patients, a significant association was found between higher natamycin MIC with a larger 3 month infiltrate/scar size and increased corneal perforations. In the results of MUTT II, the authors further found that the positive results of repeated cultures for FK patients had a poor prognosis. Patients who examined positive in their 6 day culture had twice the risk of corneal perforation or the need for therapeutic penetrating keratoplasty than those who examined negative. These patients also had worse visual acuity at 3 months. In vitro susceptibility testing of fungal pathogens is becoming increasingly important due to the frequency and diversity of FK and because resistance profiles are species-specific [69]. Reference methods for antifungal susceptibility testing are based on the standards of the Clinical and Laboratory Standards Institute (CLSI) and European Committee on Antimicrobial Susceptibility (EUCAST), but breakpoints have not yet been established. For more rapid and effective selection of antifungal agents to preserve vision and minimize the need for surgery, antifungal susceptibility tests are shifting toward omics approaches from standardized reference methods in CLSI and EUCAST, as well as commercialized methods, such as Sensititre YeastOne and Etest.

Following the advent of MALDI-TOF MS, this technique has an opportunity to identify microbial species with high rapidity and reliability. However, unlike bacteria, the identification of fungal species by MALDI-TOF MS has not yet been accepted as routine identification practice for a microbial laboratory [13]. The complexity of fungal cells, life history, and dimorphic phenotype for some fungi prevent early standardized identification procedures isolated from clinical specimens. After the minimal profile change concentration (MPCC) was introduced as a new endpoint for antifungal susceptibility [70], composite correlation index (CCI) analysis was used to obtain the linkage of mass spectra of fungal cells after a certain period of exposure to different concentrations of antifungal agents [71]. MPCC represents the CCI value at which a spectrum is more like the spectrum observed at the maximal antifungal concentration than the spectrum observed at the null antifungal concentration. Alternatively, resistant, intermediate resistant, and sensitive fungal species for an antifungal agent pre-specified by standardized reference methods were used to obtain the CCI value by matching the spectra of these species [72]. The antifungal resistance for a fungal species was then determined by directly matching the spectra without exposure to antifungal agents. Despite shortening the identification time, proteomic susceptibility tests cannot be directly applied to primary clinical samples and that culture is still needed to obtain enough fungal cells. Identification of antifungal genes and introduction to genomic or metagenomic approaches will be the next step for the direct application of clinical samples with clinically suspected FK.

## 9. Perspectives of an Omics Approach in FK

From this review, we can understand that omics approaches still have significant room for improvement in diagnosing FK, monitoring the interaction between hosts and pathogens, guiding the treatment of FK, and evaluating the treatment effects in FK events. Currently, a genomic approach for diagnosing FK may not guarantee absolute accuracy to species-level identification because most of the diagnostic methods focus on one DNA fragment, such as rRNA genes and ITSs. However, following technical advancements of next generation sequencing and the accumulation of whole genome sequencing databases, simultaneous multi-fragment amplification and detection will be possible by DNA-based molecular techniques for rapid and precise species-level identification with the coverage of critical anti-fungal genes. High-throughput sequencing techniques, including shot-gun and target-enrichment sequencing, will be powerful metagenomic solutions for elucidating the fungal pathogen–microbiota and pathogen–patient relationships during FK events, respectively. In addition, the personalized tear proteomics using MOLDI-TOF-MS and LC-MS techniques will help physicians to precisely monitor expressions of virulent factors from fungal pathogens and the defense response of patients.

Among the predictors of medical treatment failure, positive fungal culture results, day 6 repeated fungal culture positive results, *Aspergillus* species isolation, and MIC to natamycin lead to worse outcomes, and a lower threshold for surgical intervention should be adopted. Genomic and metagenomic approaches may provide alternative diagnoses for FK patients with false negative results in conventional approaches. In addition, the ability to identify fungal species with a higher risk of antifungal resistance can help physicians determine whether to adopt surgical intervention early-on or implement a more aggressive medical treatment. A metagenomics approach for monitoring pathogenic conjunctival flora in FK may help guide the antibiotic regimen to prevent bacterial superinfection. The insidious course of FK may cause physicians to overlook the disease progression of FK. A tear proteomics approach for identifying up-regulated and down-regulated proteins released from ocular surface or fungal pathogens could help physicians to determine the stage of fungal infection and the response of ocular surface immunity.

In the cloud era, different types of clinical information (risk factors and clinical photos) and laboratory data (smears, cultures, and omics surveys) from an increasing amount of FK cases will be collected more efficiently. Data mining for FK cases will help physicians develop a prognosis prediction model and evolve an optimal decision model to guide FK treatment. Following the advancement of deep learning algorithms, artificial intelligence (AI) may provide an AI-based diagnosis for inexperienced clinicians to make a near-expert diagnosis after training with a large amount of tandem information from clinical and laboratory data. Therefore, we believe a pan-omics approach will ultimately assist physicians to implement precision medicine in routine practices for FK patients in the near future.

## Figures and Tables

**Table 1:** Omics approach for diagnosing fungal keratitis.

Eyes (*n*)	Subjects *^a^*	Detection Target	Technique *^b^*	Pathogen Identification *^c^*		Reference
				Sensitivity	Specificity	
Genomics						
30	CSMK	18S rRNA gene	Nested PCR	94% (15/16)	50% (7/14)	Gaudio et al. [42]
32	CSFK	ITS2−5.8S rRNA gene	Nested PCR	93% (14/15)	24% (4/7)	Ghosh et al. [43]
30	CSMK	18S rRNA gene	PCR	88% (10/11)	82% (18/19)	Embong et al. [44]
66	CSFK	18S rRNA gene	PCR	94% (29/31)	17% (6/35)	Kim et al. [45]
40	CSFK	28S rRNA gene	PCR	70% (7/10)	57% (17/30)	Vengayil et al. [31]
28	CSFK	18S rRNA gene	Nested PCR	81% (13/16)	33% (4/12)	Badiee et al. [46]
39	CSMK	ITS2 & EF1-alpha gene, Cap5G & Cap8G gene, LytA gene, gyrB gene, mecA gene	Real-time PCR	100% (3/3)	94% (34/36)	Itahashi et al. [47]
20	DFK	ITS1-5.8S rRNA gene-ITS2	Semi-nested PCR	92.6% (25/27)	N.A.	Ferrer et al. [48]
38	CSMK	18S rRNA gene	Real-time PCR-HRM	100% (10/10)	75% (21/28)	Goldschmidt et al. [49]
50	CSMK	18S rRNA gene-ITS1-5.8S rRNA gene-ITS2-28S rRNA gene	DHA	100% (20/20)	97% (29/30)	Kuo et al. [50]
67	CSMK	ITS1-5.8S rRNA gene-ITS2	Direct PCR	96% (23/24)	24% (9/38)	Zhao et al. [51]
42	CSFK	Mitochondrial cytochrome b gene	Multiplex PCR	88% (37/42)	N.A.	He et al. [52]
559	CSFK	18S rRNA gene-ITS1-5.8S rRNA gene-ITS2-28S rRNA gene	Multiplex PCR	89% (423/473)	N.A.	Manikandan et al. [53]
Metagenomics						
1	CSFK	Tagged DNA-DNA fragment-tagged DNA	PCR, Shot-gun sequencing	N.A.	N.A.	Shigeyasu et al. [54]
18	FK	V3-V4 region of 16S rRNA gene	PCR, 16S rDNA sequencing	N.A.	N.A.	Ge et al. [55]
63	FK	ITS2−5.8S rRNA gene, V3-V4 region of 16S rRNA gene	PCR, ITS2 & 16S rDNA sequencing	N.A.	N.A.	Kalyana Chakravarthy et al. [56]
Proteomics						
56	FK	Tear proteins	2D-PAGE, MALDI-TOF MS	N.A.	N.A.	Ananthi et al. [38]
88	*Fusarium* keratitis	Tear proteins	2D-DIGE, LC-MS/MS	N.A.	N.A.	Ananthi et al. [39]
16	*Aspergillus* keratitis	Tear proteins	1D-PAGE, in-gel digestion, LC-MS/MS	N.A.	N.A.	Kandhavelu et al. [57]

*^a^*CSMK = clinically suspected microbial keratitis; CSFK = clinically suspected fungal keratitis; DFK = definite fungal keratitis; *^b^* PCR = polymerase chain reaction; HRM = high resolution melting analysis; DHA = dot hybridization assay; 2D-PAGE = two-dimensional polyacrylamide gel electrophoresis; MALDI-TOF MS = matrix-assisted laser desorption/ionization-time of flight mass spectrometry; 2D-DIGE = two-dimensional difference gel electrophoresis; LC-MS/MS = Liquid chromatography-tandem mass spectrometry; *^c^* Diagnostic standard based on microbial culture or DNA sequencing; N.A. = not available; rRNA = ribosomal RNA; ITS = internal transcribed spacer.

## Abbreviations

FK	fungal keratitis
MIC	minimal inhibitory concentration
BK	bacterial keratitis
NIKBUT	non-invasive keratographic break-up time
rRNA	ribosomal RNA
ITS	internal transcribed spacer
PCR	polymerase chain reaction
PCR-HRM	real-time PCR with high resolution melting analysis
DHA	dot hybridization assay
OTU	operational taxonomic unit
2D-PAGE	two-dimensional polyacrylamide gel electrophoresis
MALDI-TOF MS	matrix-assisted laser desorption/ionization-time of flight mass spectrometry
2D-DIGE	two-dimensional difference gel electrophoresis
LC-MS/MS	liquid chromatograph coupled tandem mass spectrometry
MUTT	Mycotic Ulcer Treatment Trial
CLSI	Clinical and Laboratory Standards Institute
EUCAST	European Committee on Antimicrobial Susceptibility Testing
MPCC	minimal profile change concentration
CCI	composite correlation index
